# Genetic Factors Associated with Clinical Response in Melanoma Patients Treated with Talimogene Laherparapvec: A Single-Institution Retrospective Analysis

**DOI:** 10.1245/s10434-024-16346-x

**Published:** 2024-10-18

**Authors:** Kailan Sierra-Davidson, Aikaterini Dedeilia, Aleigha Lawless, Tanya Sharova, Howard L. Kaufman, Genevieve M. Boland, Sonia Cohen

**Affiliations:** Department of Surgery, Massachusetts General Hospital, Harvard Medical School, Boston, MA USA

**Keywords:** Melanoma, Oncolytic viruses, Talimogene laherparepvec, TERT, Head and neck cancers, SNaPSHOT genetic testing

## Abstract

**Background:**

Talimogene laherparapvec (T-VEC) is a modified herpes simplex virus type 1 (HSV-1) and the first oncolytic virus to be approved for the treatment of unresectable melanoma. We assessed whether there are tumor-intrinsic genetic factors that are associated with tumor control.

**Methods:**

A single-institution, retrospective analysis of melanoma patients treated with T-VEC was performed. Demographics, histopathologic reports, treatment history, clinical outcomes, and tumor genomic analysis of approximately 100 genes were collected.

**Results:**

Ninety-three patients who had received T-VEC were identified, of whom 84 (91%) were diagnosed with cutaneous melanoma. Sixty-nine (69) patients received more than one dose of T-VEC and had sufficient data available for clinical analysis. Of these patients 30.0% (*n* = 21) had evidence of a complete response, defined as complete regression of all lesions without the need for additional treatment or procedures. Stage III disease (*p* < 0.001), absence of macroscopic nodal disease (*p* < 0.001), and absence of visceral/central nervous system metastases (*p* = 0.004) were all associated with evidence of any clinical response or local control by univariate analysis. At the time of analysis, 54 patients had tumor genetic data available. Sixty genes were mutated in at least one patient, and all but one patient had at least one gene mutation identified. Presence of TERT promotor mutation was associated with evidence of any clinical response (*p* = 0.043) or local control (*p* = 0.039) by multivariate analysis.

**Conclusions:**

This work describes the experience using T-VEC in melanoma at a single institution and highlights the presence of TERT promotor mutations as a possible driver of clinical response.

**Supplementary Information:**

The online version contains supplementary material available at 10.1245/s10434-024-16346-x.

Melanoma remains one of the most common cancers, with approximately 100,640 new cases and 8290 deaths expected in 2024.^1^ While surgical resection with wide local excision (WLE) remains essential for curative treatment, advances in medical treatments with immune checkpoint blockade (ICB), adoptive T-cell therapy, and targeted therapies with BRAF/MEK inhibitors have revolutionized melanoma management.^2–4^ Localized treatment with oncolytic viruses have provided an additional but understudied tool for unresectable primary tumors.^5^ The optimal use of this emerging class of treatment remains unclear.

Talimogene laherparapvec (T-VEC) is currently the only oncolytic virus to be approved for the treatment of melanoma in the United States (US) and consists of a modified herpes simplex virus type 1 (HSV-1) expressing granulocyte-macrophage colony-stimulating factor (GM-CSF). US FDA approval in 2015 was based on findings from the OPTiM phase III trial that demonstrated improved durable response rates in patients with stage IIIB-IVM1C unresectable melanoma who received intralesional T-VEC (16.3%) compared with GM-CSF (2.1%).^6,7^

Additional randomized trials have looked to further define the clinical indications for T-VEC in the current melanoma treatment landscape. One promising strategy is neoadjuvant therapy. In a recent phase II clinical trial, patients with resectable IIIB-IVM1a disease who received neoadjuvant T-VEC followed by surgery had a 25% reduction in disease occurrence at 2 years compared with patients who received upfront surgery.^8,9^ Another recent randomized phase III study evaluated the use of T-VEC in combination with pembrolizumab. The Masterkey-265/KEYNOTE-034 study evaluated pembrolizumab with and without T-VEC in checkpoint inhibitor-naïve patients with unresectable stage IIIB-IVM1c melanoma.^10^ While there was no significant difference in progression-free survival (PFS) or overall survival (OS) between the cohorts, it is noteworthy that overall, the patients in this trial had more advanced disease compared with the original OPTiM study. Approximately 41% of patients included in Masterkey-265 had M1c disease,^10^ in contrast to the OPTiM study, which excluded patients with extensive visceral disease.^6^ These clinical trials suggest that disease stage may play an important role in predicting treatment response, but the optimal patient and tumor characteristics to select patients for treatment remain undefined.

Predictive genetic biomarkers of response to oncolytic virus therapy would be useful in selecting patients who would best benefit from T-VEC therapy. While there has been limited work in this field, small *in vitro* studies have suggested that intracellular antiviral pathways may be critical in T-VEC efficacy. For example, loss-of-function mutations in the interferon (IFN) JAK-STAT signaling pathway and low STING expression in melanoma cells are associated with increased sensitivity to oncolytic viruses;^11,12^ however it remains unknown if there are additional genetic markers that could be used to select patients who would best respond to T-VEC therapy. While there have been a number of retrospective analyses that have looked at the use of T-VEC in the real-world,^13–23^ tumor genetic analysis in these studies is limited. As tumor-intrinsic characteristics may drive patient responses to T-VEC, we set out to explore the clinical characteristics and mutational burden of tumors using gene panel testing (SNaPSHOT) in patients who have been treated with T-VEC as part of standard of care at a large academic referral center.

## Methods

### Patient Selection

We performed a retrospective analysis of patients who received at least one dose of T-VEC for treatment of cutaneous melanoma at our institution. Patients with cutaneous melanoma were staged according to the Eighth Edition of the American Joint Committee on Cancer (AJCC) criteria. Patients were selected from a large melanoma cohort study and tumor biobank maintained at Massachusetts General Hospital for more than 14 years (2009–2023). The study was approved by the Institutional Review Board. Patients were treated with T-VEC at 1 × 10^6^ plaque-forming units (PFUs)/milliliter (mL) by intra-tumoral injection as a priming dose and then 3 weeks later they received T-VEC at 1 ×10^8^ PFU/mL by intra-tumoral injection every 2 weeks. Whole-body imaging was generally performed every 12 weeks unless otherwise indicated.

### Inclusion and Exclusion Criteria

Demographics and clinical outcomes were assessed in all patients who were diagnosed with cutaneous melanoma and who received a full dose of T-VEC. Patients with squamous cell carcinoma, mucosal melanoma, or uveal melanoma were excluded from the analysis, given the distinctive molecular profiles and prognosis. Additionally, patients who stopped treatment after the test dose due to either logistical reasons or transition to hospice were also excluded from the study. Acral lentiginous melanomas were included in the study and noted. Prior diagnoses of melanoma in situ, basal cell carcinoma, or squamous cell carcinoma did not exclude patients from this study. Four patients who met the inclusion and exclusion criteria but were still receiving active T-VEC therapy were excluded as it was too early to determine therapeutic outcome at the time of final statistical analysis. For the tumor profiling analysis, all patients in the first part of the study who had SNaPSHOT genomic analysis were included.

### Data Extraction

Briefly, we extracted general demographic information, histopathologic reports, additional systemic treatments, details of T-VEC treatment course, clinical response to T-VEC, and tumor genetic information for each patient. The medical record review and data extraction occurred from August 2023 through February 2024, and statistical analysis occurred in March 2024. The demographic data extracted were name, date of birth, sex, and age at the time of T-VEC treatment, as well as date and cause of death, if applicable. The clinical information extracted was location of the primary tumor, date of biopsy and/or WLE if applicable, pathology details (including Breslow thickness, mitotic rate, and presence of ulceration, lymphovascular invasion, and tumor-infiltrating lymphocytes), status of sentinel lymph node biopsy (SLNB) and/or complete lymph node dissection (CLND) if applicable, tumor-node-metastasis (TNM) staging (according to the 8th Edition AJCC criteria) at the time of T-VEC treatment, pre- and post-T-VEC systemic therapy regimen(s), and concurrent therapy regimen(s), if applicable. We also assessed the presence of in-transit metastases, bulky nodal disease, and presence of visceral/brain metastases at the time of T-VEC treatment. Per our practice, all dermal-based metastases are called ‘in-transit metastases’ and all subcutaneous lessions are called ‘soft tissue metastases’. Information on the T-VEC treatment course extracted included injection sites and dates, number of courses, number of total doses, and pathology from post-treatment biopsies of injected sites if available.

Response to treatment was assessed and divided into four categories: complete response (CR), mixed local response (MLR), partial response with local control but distant progression of disease (LCDP), or no response (NR). Based on these categories, the two dichotomous variables of any response (categories CR/MLR/LCDP vs. NR) and of local response (categories CR/LCDP vs. NR) were calculated as the two endpoints of interest.

Tumor genetic information was also extracted for available patients. Tumor genomic analysis had previously been performed for clinical purposes using the SNaPSHOT multiplexed polymerase chain reaction (PCR) assay. This assay was performed through the MGH Center for Integrated Diagnostics using formalin-fixed, paraffin-embedded tissue. The current version of SNaPSHOT assesses single nucleotide variants (SNVs), insertions or deletions (indels), and copy number variants (CNVs) in approximately 100 genes. Tumor mutational burden (TMB) was not available for all patients, and thus surrogate TMB was calculated as number of mutated genes detected by the SNaPSHOT assay. The specific mutations found in the most commonly mutated genes were also assessed.

### Statistical Analysis

Statistical analysis was performed using IBM SPSS Statistics for Windows, version 28.0.0.0 (IBM Corporation, Armonk, NY, USA). Statistical significance was set at *p* < 0.05. All continuous variables were reported as median (interquartile range [IQR]) and all categorical variables were reported as frequency and percentage (%). To assess the effect of demographic, histopathological, and tumor genetic factors on the two study endpoints (total response and local response) that constituted binary outcomes (response or non-response), univariate binary logistic regressions were applied. All factors that were statistically significant in the univariate analysis were assessed for multicollinearity using the Variance Inflation Factor (VIF) test. A VIF value of ≥ 4 was used to define collinearity. Colinear variables were excluded from the analysis prior to initiation of stepwise regression. To assess the independent effect of all remaining significant variables identified from the univariate analyses, multivariable binary logistic regression models were implemented with an automatic stepwise addition of each variable. The inclusion criteria for addition of a factor at each step required a *p* value of <0.05. The stepwise regression concluded when the addition of any remaining variables to the model did not improve the model. The final multivariate models had a *p* value of <0.001. Forest plots were created to depict the odds ratios (OR) of statistically significant univariate and multivariable logistic regression models.

## Results

**Fig. 1:**
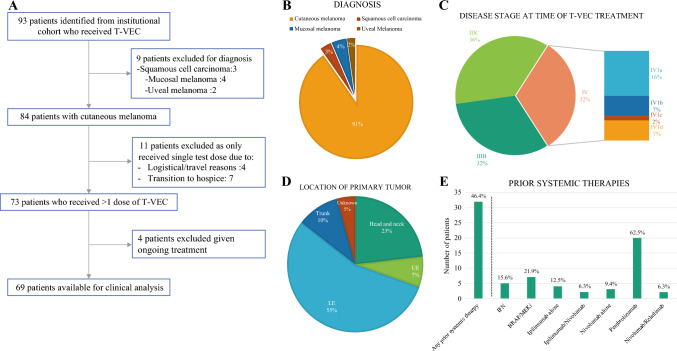
Characteristics of the study cohort. **A** Patients identified at this institution, with key inclusion and exclusion criteria. **B** Histologic diagnosis of all patients treated at this institution who received at least one dose of T-VEC (*n* = 93). **C** Disease stage at the time of T-VEC treatment, according to the AJCC 8th edition, of all patients available for clinical outcomes analysis (*n* = 69). **D** Location of the primary tumor of all patients available for clinical outcomes analysis (*n* = 69). Lower extremity patients include those with acral lentiginous melanoma, while head and neck patients include those with scalp, lower neck, and post-auricular tumors. **E** Prior systemic therapies of all patients available for clinical outcomes analysis (*n* = 69). Percentage to the left of the dashed line reflects the percentage of patients who received systemic therapy among all patients in the cohort, while percentages to the right of the dashed line reflect the percentage of patients who received each specific treatment among those who received systemic therapy. Note that patients may have received more than one type of prior treatment. *BRAF/MEKi* BRAF/MEK inhibitors, *IFN* interferon, *UE* upper extremity, *LE* lower extremity, *T-VEC* talimogene laherparepvec, *AJCC* American Joint Committee on Cancer

From our single-institution cohort, 93 patients who had received T-VEC were identified, of whom 84 (91%) were diagnosed with advanced cutaneous melanoma (Fig. 1A, B). The remaining diagnoses included squamous cell carcinoma (*n* = 3), mucosal melanoma (*n* = 4), and uveal melanoma (*n* = 2). These patients were excluded from the analysis due to the unique biology of these tumors. Eleven (11) patients stopped treatment early after a single test dose due to logistical/travel reasons (*n* = 4) or decision to transition to hospice (*n* = 7). Four patients were currently being treated and did not have sufficient information available to determine clinical outcome. Overall, there were 69 patients with cutaneous melanoma with sufficient information for outcomes analyses.

We first assessed the study cohort as a whole. Demographic information is summarized in Table 1. Among all patients, 31.9% (*n* = 22) had stage IIIB disease at the time of T-VEC treatment, 36.2% (*n* = 25) had stage IIIC disease, and 31.9% (*n* = 22) had stage IV disease (Fig. 1C). Primary tumor location was varied, with the two most common locations being the lower extremities (55%, *n* = 38) and head and neck (23%, *n* = 16) [Fig. 1D]. Head and neck patients included patients with scalp (*n* = 14), lower neck (*n* = 1), and post-auricular (*n* = 1) tumors. In order to further characterize the extent of disease and understand the clinical reasoning for T-VEC use, we assessed patients for the presence of in-transit metastases, macroscopic nodal disease (as defined by either clinically palpable nodes or ^18^F-fluorodeoxyglucose [FDG]-avid, biopsy-proven nodes), and visceral and/or central nervous system (CNS) metastases. In our cohort, 88.4% (*n* = 61) of patients treated with T-VEC had in-transit metastases, while only 15.9% (*n* = 11) had visceral or CNS metastases (Table 1). Regarding prior treatment history, 46.4% (*n* = 32) had received prior systemic therapy, of whom 84.3% (*n* = 27) had previously been treated with checkpoint inhibitors (Fig. 1E, Table 2). Among those previously treated with checkpoint inhibitors, 33% (*n* = 9) had been treated in the adjuvant setting and the remainder were treated for unresectable stage III or stage IV disease. In terms of T-VEC treatment, patients in our cohort received a median of 5 doses (range 2–27), with 7.8% (*n* = 4) of patients receiving multiple courses and 31.4% (*n* = 16) receiving concurrent systemic therapy.

**Table 1:** Patient demographics

Characteristics	Total patients (*n* = 69)
Sex	
Male	37 (53.6%)
Female	32 (46.7%)
Age	
Median age (range)	78 (25-96)
< 60	6 (8.7%)
60–80	34 (49.3%)
> 80	29 (42.0%)
Disease stage (AJCC 8th edition)—no. (%)	
IIIB	22 (31.9%)
IIIC	25 (36.2%)
IV1a (skin, subcutaneous or nonregional nodes)	11 (15.9%)
IV1b (lung)	5 (8.3%)
IV1c (non-CNS visceral)	1 (1.4%)
IV1d (CNS)	5 (8.3%)
Tumor location—no. (%)	
Head and neck	16 (23.1%)
Upper extremity	5 (7.2%)
Lower extremity	38 (55.1%)
Trunk	7 (10.1%)
Unknown	3 (4.3%)
Extent of disease—no. (%)	
In-transit metastases	61 (88.4%)
Macroscopic nodal disease	26 (37.7%)
Visceral or CNS metastases	11 (15.9%)

**Table 2:** Univariate and multivariable logistic regressions

Univariate							Multivariable					
	Odds ratio	Lower 95% CI	Upper 95% CI		*p* value				Odds ratio	Lower 95% CI	Upper 95% CI	*p* value
(A) *Any kind of response to TVEC*												
Clinical evidence of nodal disease	0.135	0.045	0.402		< 0.001 ***		ns					
Stage III or IV	0.129	0.041	0.404		< 0.001 ***		Stage III or IV		0.108	0.020	0.596	0.011*
Disease stage (IIIB, IIIC, IVA/B/C/D)	0.405	0.24	0.682		< 0.001 ***		n/a (collinear)					
Number of TVEC doses	1.397	1.135	1.719		0.002**		Number of TVEC doses		1.427	1.104	1.845	0.007 **
Brain/Visceral mets	0.045	0.005	0.379		0.004**		n/a (collinear)					
Concurrent systemic therapy	0.328	0.108	0.998		0.050~		ns					
Prior systemic therapy	0.373	0.139	1.004		0.051~		ns					
TERT mutation	3.515	0.991	12.464		0.052~		TERT mutation		6.140	1.076	35.026	0.041*
(B) *Local response to TVEC*												
Clinical evidence of nodal disease	0.107	0.031	0.366		<0.001 ***		ns					
Brain/Visceral mets	0.060	0.007	0.508		0.010*		n/a (collinear)					
Stage III or IV	0.144	0.043	0.486		0.002**		Stage III or IV		0.079	0.011	0.548	0.010*
Disease stage (IIIB, IIIC, IVA/B/C/D)	0.427	0.25	0.73		0.002**		n/a (collinear)					
Number of TVEC doses	1.394	1.12	1.736		0.003**		Number of TVEC doses		1.462	1.120	1.908	0.005 **
Prior systemic therapy	0.308	0.106	0.897		0.031*		ns					
TERT mutation	5.818	1.29	26.249		0.022*		TERT mutation		9.811	1.298	74.131	0.027*

**Fig. 2:**
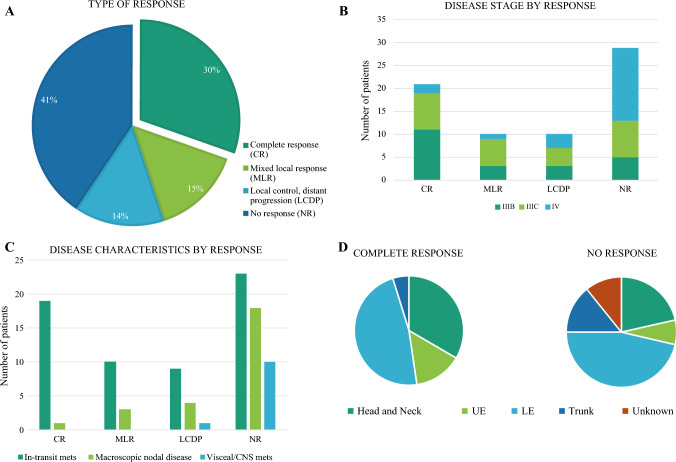
Study cohort by response. **A** Pie chart of all patients available for outcomes analysis, broken up by clinical response (*n* = 69). **B** Disease stage according to the AJCC 8th edition, by clinical response. **C** Disease characteristics, by clinical response. Note that patients could have more than one disease characteristic (i.e. in-transit metastases and macroscopic nodal disease). **D** Tumor location of patients with complete response (*n* = 21) versus no response (*n* = 28). *UE* upper extremity, *LE* lower extremity, *CR* complete response, *MLR* mixed local response, *LCDP* local control/distant progression, *NR* no response, *AJCC* American Joint Committee on Cancer, *CNS* central nervous system metastases, *mets* metastases

Clinical response to treatment with T-VEC at the time of chart extraction was then evaluated in this cohort. The median follow-up time from initial T-VEC treatment to death or last clinic visit was 14 months (range 3–60 months). In order to assess the effect of T-VEC on both local injected lesions and distant disease, we categorized patient responses into four different cohorts. Of the 69 patients in our cohort, 30.4% (*n* = 21) had evidence of a CR, defined as complete regression of all lesions without the need for additional treatment or procedures (Fig. 2A). Based on the Response Evaluation Criteria in Solid Tumors (RECIST) 1.1 criteria, 29.0% (*n* = 20) had a best objective response (BOR) of a partial response (PR). Twenty-eight patients (40.6%) had no evidence of clinical response to T-VEC (NR). There were no patients in our cohort who had stable disease at the timepoint captured. In order to better understand the effect of local therapy on local lesions, we further divided the patients with PR. Ten patients (14.5%) had regression of some injected cutaneous lesions with progression of others, requiring either additional surgery or systemic treatment, labeled as an MLR. Ten patients (14.5%) had complete regression of injected cutaneous lesions but development of distant metastases, suggestive of a complete local response with distant progression (local control/distant progression; LCDP).

We then investigated the clinical drivers of a CR. Of those patients who had a CR, 19/21 had stage IIIB/C disease (Fig. 2B, electronic supplementary material [ESM] Table 1). The two remaining complete responders had stage IVM1A, defined as metastases to distant subcutaneous lesions or non-regional lymph nodes. Thus, there were no patients with visceral or CNS disease who showed a CR to T-VEC, even in the setting of concurrent anti-programmed death-1 (PD-1) therapy (Fig. 2C). In terms of clinical indications in the complete responder group, 3/21 patients received T-VEC as upfront treatment (i.e., they had received no prior therapy) and 18/21 patients were treated for re-presentation with in-transit metastases (ESM Table 2). Interestingly, 5/21 patients with a CR received concurrent systemic therapy, including BRAF/MEK inhibitors, pembrolizumab, or nivolumab/relatlimab.

Subset analysis of the OPTiM trial noted a higher response rate to T-VEC compared with GM-CSF among patients with head and neck melanomas.^6^ We thus assessed if tumor location was a driver of clinical response in our cohort. There was no clear pattern of tumor location in patients with CR versus NR (Fig. 2D, ESM Table 3). Notably, the response rate among patients with head and neck tumors in our cohort may have been driven by disease stage. Of the seven patients with head and neck tumors who had CR, six had stage III disease. In contrast, only two of the six patients with head and neck tumors who had NR had stage III disease (ESM Fig. 1). Additionally, there was an overall poor response among our small cohort of patients with acral lentiginous melanoma (*n* = 3), as two patients had no evidence of response and one patient had an MLR.

**Fig. 3:**
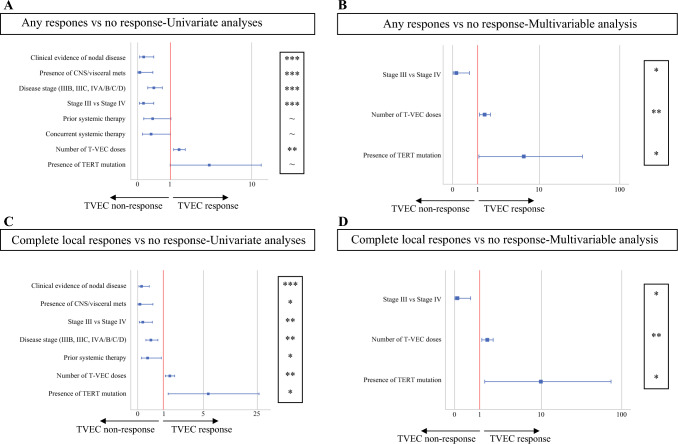
Forest plots of drivers of response to T-VEC treatment. Forest plots of Cox regression hazard ratios among patients with any response (CR/MLR/LCDP) versus NR (**A, B**) and local control (CR/LCDP) versus NR (**C, D**). Univariate analysis on the left panel (**A, C**) includes all variables assessed. Multivariate analysis on the right panel (**B, D**) includes only significant, non-overlapping variables from univariate analysis. Presence of TERT gene mutation is included in multivariate as this was the only significant genetic factor in univariate analysis. The analysis included 69 patients for histopathological and clinical factors and 54 patients for genetic factors. ~*p* = 0.05–0.06, *p = 0.01–0.05, **p = 0.001–0.01, ***p < 0.001. *CR* complete response, *MLR* mixed local response, *LCDP* local control/distant progression, *NR* no response, *T-VEC* talimogene laherparepvec, *CNS* central nervous system, *mets* metastases

We performed univariate and stepwise multivariable logistic regression analysis of clinical findings described above, focusing on statistically significant drivers of any clinical response versus NR, as well as complete local response versus no local response. We hypothesized that stage and extent of disease would be key determinants of response (Fig. 3). Based on univariate analysis, stage III disease (*p* < 0.001), absence of macroscopic nodal disease (*p* < 0.001), and absence of visceral/CNS metastases (*p* = 0.004) were all associated with evidence of any clinical response. The variables of stage and absence of visceral/CNS metastases were colinear in this new model, therefore only stage (III vs. IV) was included in the stepwise multivariable analysis. Stage III versus IV remained statistically significant in the multivariable logistic regression models for both any response (*p* = 0.011) and local control of disease (*p* = 0.010). Prior systemic therapy (*p* < 0.05) and concurrent therapy (*p* < 0.05) were also associated with NR by univariate analysis but these results were not significant and were thus not included in the multivariable analysis, possibly reflecting the use of TVEC as a last resort therapy. Finally, the total number of T-VEC doses was associated with any response as well as with local response in both univariate and multivariate models, possibly because patients with an ongoing encouraging clinical response often continue treatment.

**Fig. 4:**
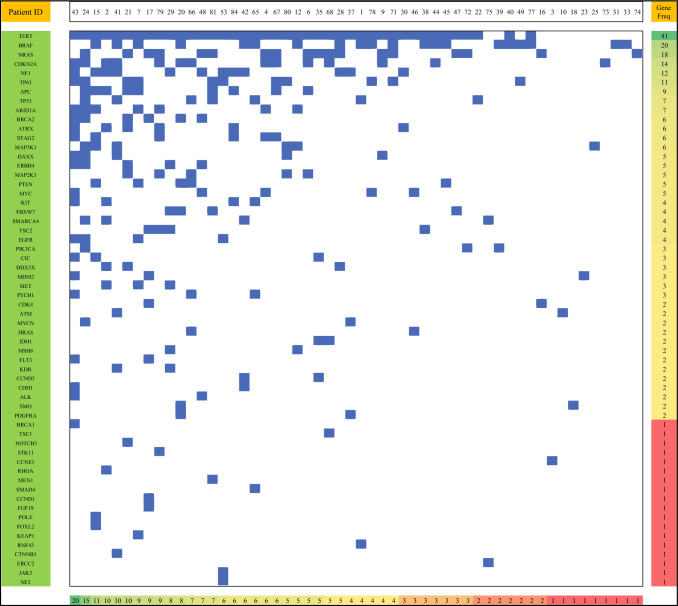
Heat map of the most commonly mutated genes, as identified by the SNaPSHOT assay. All genes that were mutated in at least one patient are ordered by frequency across the cohort (*n* = 60 genes). De-identified patient ID is shown on the top panel. Patients are ordered by number of gene mutations identified (bottom panel)

SNaPSHOT data were available in 54 patients (ESM Table 4). Sixty genes were mutated in at least one patient, and all but one patient had at least one gene mutation identified. Patients had a median of 3 gene mutations identified (range 0–41). The most commonly mutated genes were TERT, BRAF, NRAS, and CDKN2A, consistent with known patterns of mutations in cutaneous melanoma. The heatmap in Fig. 4 depicts all gene mutations identified, ordered by prevalence of mutations across the entire cohort*.*

**Fig. 5:**
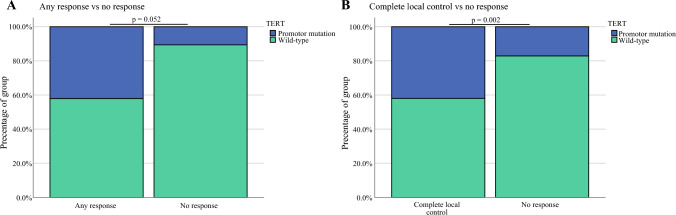
Patients with evidence of any response or local control by TERT gene expression. Presence of TERT promotor mutation or wild-type gene among patients with **A** any response (CR/MLR/LCDP) versus NR; and **B** local control (CR/LCDP) versus NR. *P* values are based on univariate analysis. *CR* complete response, *MLR* mixed local response, *LCDP* local control/distant progression, *NR* no response

We then performed univariate analysis of mutated genes with a similar grouping strategy as above, focusing on statistically significant drivers of any clinical response (CR/MLR/LCDP vs. NR) or local response (CR/LCDP vs. NR). Based on univariate analysis, the only gene mutation that was statistically significant was TERT. Presence of a TERT mutation was associated with evidence of any clinical response (*p* = 0.052), as well as evidence of local control (*p* = 0.022) compared with NR (Fig. 5). When included in a multivariate analysis with the statistically significant clinical drivers (Fig. 3), the presence of a TERT mutation remained statistically associated with evidence of any response (*p* = 0.041) and local response (*p* = 0.027). In our cohort, TERT mutations were most commonly SNVs (*n* = 35), in addition to CNV gain (*n* = 2) and indels (*n* = 3). All of these mutations were found in the promotor region, with SNVs as either hotspot C228T or C250T variants. There was no statistically significant pattern of TERT mutations across the clinical groups. No additional gene mutations were statistically significant.

TMB was only included in a subset of SNaPSHOT reports, and thus a TMB surrogate was assessed, defined as the number of gene mutations identified by the SNaPSHOT report. The TMB surrogate was not associated with any evidence of any or local response to T-VEC therapy by univariate or multivariate analysis.

## Discussion

Given the increasing use of oncolytic viruses in clinical practice, better understanding of which patients would benefit from such therapy is needed. This retrospective analysis assessed cutaneous melanoma patients from a single institution in order to investigate clinical and tumor genetic factors that may be associated with response to T-VEC. In our cohort, 30.0% (*n* = 21) had evidence of CR and 44.9% (*n* = 31) had evidence of compete local control (CR + LCDP). These data are similar to the response rate from a similar-sized multi-institutional retrospective study.^14^ We hypothesized that earlier disease stage and a lower burden of disease would be associated with treatment response to T-VEC. We showed that stage III disease, absence of macroscopic nodal disease, and absence of visceral/CNS metastases were significantly associated with any clinical response and local disease control in univariate but not multivariate analysis. This may perhaps reflect the relatively small cohort size. Additionally, we were able to take advantage of genetic panel testing using our institutional genomic SNaPSHOT assay in order to assess the impact of mutations in approximately 100 genes on the efficacy of T-VEC. We found that TERT promotor mutations were associated with any clinical response and local control in both univariate and multivariate analysis in melanoma patients treated with T-VEC. This study builds on prior retrospective studies examining real-world use of T-VEC and suggests that intrinsic tumor factors may impact response to oncolytic viral therapies.

Melanoma stage and extent of disease have previously been observed as important factors in response to T-VEC therapy.^7,13,14,24,25^ In the OPTiM trial, subgroup analyses demonstrated that patients with stage IIIB-IVM1a disease according to the AJCC 7th edition had improved durable response rates at 5 years compared with all patients (hazard ratio 0.56, 95% confidence interval 0.40–0.79, *p* = 0.008).^7^ Additionally, a recent retrospective analysis of 80 melanoma patients treated with T-VEC showed that patients with stage IIIB disease were more likely to have a complete local response (68%) compared with stage IIIC, IIID, and IV disease (26%, 0%, and 6%, respectively) based on AJCC 7th edition staging.^14^ It is possible that our study was underpowered to assess this finding, which may explain why we observed stage III disease, absence of macroscopic nodal disease, and absence of visceral/CNS metastases as significant variables associated with response in univariate but not multivariate analysis. In our population, it was also notable that over 88% had in-transit metastases, suggesting that T-VEC may be especially useful for management of in-transit melanoma metastases. Additionally, it is also notable that our study cohort includes patients who received T-VEC as upfront treatment without systemic therapy, including patients with isolated scalp lesions.

Overall, our study may further support the hypothesis that the absence of clinical improvement of combination therapy with pembrolizumab and T-VEC in Masterkey-265 was driven by inclusion of patients with visceral disease with greater disease burden than typically treated in the real-world. Our study and others^7,13,14^ show that disease stage may be a critical factor in determining overall patient responses to T-VEC. In light of these data, it remains an open question as to whether combination with T-VEC and ICB could improve response rates compared with ICB alone in the appropriate patient population. We recommend that future clinical trials should investigate T-VEC combination therapy in patients restricted to stage IIIB-IVM1a disease.

In categorizing clinical responses, we investigated both the effect of T-VEC on local injected lesions as well as distant progression in order to understand locoregional responses as well as a potential abscopal response. Ultimately, this was difficult to determine as 18/69 patients in our cohort were receiving concurrent systemic therapy, including the two patients with stage IVM1a disease who had a CR. While it is possible that the patients with LCDP had an incomplete response to T-VEC therapy, it is also possible that patients who had local control with distant progression had undetectable micrometastases already present at the time of intralesional therapy. It is unclear if these patients may have benefited from concurrent systemic therapy. Interestingly, a recent study looked at real-world use of T-VEC in the era of PD-1 inhibitors and saw no difference in objective response rates among patients who received T-VEC after anti-PD-1 therapy, received concurrent T-VEC and anti-PD-1 treatment, or only received T-VEC.^17^

Head and neck melanomas have been noted to have better response rates to T-VEC in subset analysis of the OPTiM trial.^6^ It has been hypothesized that head and neck tumors may be more susceptible to T-VEC due to increased TMB given high sun exposure.^26^ Our study included 23.1% (16/69) of patients with head and neck tumors, similar to prior retrospective studies.^14,16,19^ In line with these studies, we did not see any association with clinical response and head and neck tumors in our population. While it is possible that we were underpowered to see this type of response, it is also likely that responses in our cohort of head and neck melanomas was driven by stage, as 6/7 complete responders in our cohort had stage III disease and 4/6 had distant visceral metastases with stage IIIB-D disease. As recurrent disease in head and neck tumors can be challenging to treat given surgical limitations, and systemic therapy may not always be tolerated, T-VEC remains a useful treatment option for these patients.^27^

The key strength of our study over previous retrospective analyses was the inclusion of genetic panel testing. To our knowledge, the most comprehensive retrospective analysis from a molecular testing perspective only assessed mutations in a limited set of genes (*BRAF*, *NRAS*, and *C-KIT*).^19^ By contrast, our study allowed for the assessment of mutations in approximately 100 genes with SNaPSHOT testing. This genetic panel testing has been useful in previous work in supporting molecular factors for prognostication and risk stratification in melanoma patients.^28^ As expected based on prior work,^29^ the most commonly mutated genes in our cohort were TERT, BRAF, NRAS, and CDKN2A. The landscape of genetic alterations in our cohort was notable for a range of 0–41 gene mutations detected. In comparison, a recent study looking at SNaPSHOT genetic testing in high-risk stage II cutaneous melanoma patients reported a lower range of gene mutations of 0–30.^28^

Interestingly, only 37% (20/54) of patients in our cohort had a BRAF mutation, which is lower than reported in larger studies, which report a frequency of around 50% of primary melanomas.^30,31^ This may reflect a selection bias given the widespread use of BRAF/MEK inhibitors for patients with activating BRAF mutations, and a decreased need among these patients for additional treatments such as T-VEC. Indeed, three other retrospective studies of patients treated with T-VEC also reported lower frequencies of BRAF mutations compared with all melanoma patients: 35.2% (31/88),^19^ and 22.8% (19/83),^18^ 36.4% (24/66),^32^ and 22.4% (17/76).^16^

The most notable finding of our study was the association of TERT promotor mutations with evidence of clinical response as well as local disease control. The *telomerase reverse transcriptase (TERT)* gene encodes the catalytic subunit of telomerase, and increased activity of this gene plays an important role in carcinogenesis by maintaining telomere length to support chromosomal stability.^33,34^ Mutations in the promotor region of TERT are found in up to 70% of sporadic melanomas, and germline mutations in TERT can be associated with familial melanoma.^35,36^ TERT promotor mutations are thought to be an overall poor prognostic factor, as increased telomerase activity is associated with ulceration, increased mitotic rate, increased Breslow thickness, and satellite lesions.^37,38^ Additionally, TERT mutations are associated with increased TMB, neoantigen load, and decreased tumor heterogeneity across multiple cancer types.^39^ Interestingly, meta-analysis of nearly 10,000 patients across multiple cancer types demonstrated that patients with TERT mutations had immune signatures associated with lymphocyte infiltration and IFN-$\upgamma$ response.^39^ As T-VEC is hypothesized to promote preferential viral replication in tumor cells, cell lysis, and release of neoantigens,^40,41^ it is possible that tumor cells with TERT promotor mutations are more likely to generate an effective immune response. Increased neoantigen load associated with TERT mutations may broaden the anti-tumor CD8^+^ T-cell response. While we did not see any association with TMB surrogate, this measure likely does not accurately reflect the breadth of gene mutations or neoantigens that are presented on the tumor cell surface.

As SNaPSHOT and similar genetic panel testing are commonly used in tertiary health care centers, the presence of TERT mutation could be easily identified without additional testing outside of a streamlined process. Future clinical trials could include TERT mutational status as an additional molecular prognostic marker to help identify which patients would best benefit from this therapy. Additionally, *in vitro* studies could delineate how increased telomerase activity could lead to improved oncolytic viral replication. Overall, this study supports the use of molecular genetic testing in prognostication among patients treated with T-VEC.

### Study Limitations

This study was a retrospective analysis from a single tertiary institution. As such, there are inherent biases based on referral patterns and the practice models of a smaller group of clinicians. Furthermore, referrals for T-VEC therapy have likely changed over time, as clinicians gained more experience. As follow-up time is limited, it is possible that analysis at later time point would have shown that patients with a CR either recurred or developed distant disease. Due to the use of concurrent use of systemic therapy in a subset of our cohort, it is difficult to completely delinate the effect of T-VEC alone compared with T-VEC and systemic therapy. Additionally, as our cohort was limited to a single institution, we may have been underpowered to detect additional prognostic markers.

### Study Strengths

Despite these limitations, there are several strengths to this study. The key strength of our study over previous retrospective analyses was the inclusion of genetic panel testing. To our knowledge, no prior study has assessed the mutational burden of tumors in patients treated with T-VEC with this degree of breadth. Our study allowed for the assessment of mutations in approximately 100 genes using SNaPSHOT testing. Our cohort size is similar to prior retrospective studies assessing real-world use of T-VEC. We identified a potential biomarker, specifically TERT promotor mutations, which was associated with evidence of any clinical response and complete local control.

## Conclusion

This retrospective study describes the experience using T-VEC in cutaneous melanoma at a single institution and highlights the utility of including genetic panel testing in future clinical trials. We found TERT promotor mutations as a possible driver of clinical response, meriting further studies.

## Supplementary Information

Below is the link to the electronic supplementary material.

Supplementary file1 (PDF 99 kb)
