# Supplementary material for: Genetic Factors Associated with Clinical Response in Melanoma Patients Treated with Talimogene Laherparapvec: A Single-Institution Retrospective Analysis
Source: Ann Surg Oncol. 2024 Oct 18;32(1):482–94. doi: 10.1245/s10434-024-16346-x (PMC11659343; doi:10.1245/s10434-024-16346-x)

Supplementary Table 1: Disease Stage by Clinical Response

	IIIB	IIIC	IV1a	IV1b	IV1c	IV1d
CR	11	8	2	0	0	0
MLR	3	6	1	0	0	0
LCDP	3	4	2	0	0	1
NR	5	7	6	5	1	4
**Total Patients Treated**	**22**	**25**	**11**	**5**	**1**	**5**

Supplementary Table 2: Description of T-VEC Treatment by Clinical Response

	Median number of total doses (range)	Multiple courses of TVEC?	Prior wide local excision?	Prior exposure to systemic therapy?	Concurrent systemic therapy?	Received T-VEC as upfront therapy	Received T-VEC for new disease developed <8 weeks following surgery
CR	6 (3-19)	1/21	18/21	6/21	5/21	3/21	1/21
MLR	11 (3-27)	2/10	10/10	5/10	0/10	0/10	1/10
LCDP	8 (3-19)	1/10	10/10	4/10	2/10	0/10	1/10
NR	4 (2-12)	0/28	24/28	17/28	11/28	2/28	2/28
**Total Patients Treated**	**5 (2-27)**	**4/69**	**63/69**	**32/69**	**18/69**	**5/69**	**5/69**

Supplementary Table 3: Tumor Location by Clinical Response

	Head and Neck	UE	LE	Trunk	Unknown
CR	7	3	10	1	0
MLR	2	0	7	1	0
LCDP	1	0	8	1	0
NR	6	2	13	4	3
**Total Patients Treated**	**16/69**	**5/69**	**38/69**	**7/69**	**3/69**

Supplementary Table 4: Tissue Origin of SNAPSHOT Data

Tissue Origin	Number of Patients
Primary tumor	11
In transit metastases	32
Metastatic lymph node	6
Distant soft tissue metastases	5
**Total Patients with SNAPSHOT Data Available**	**54**

Supplementary Figure 1: Clinical Response of Head and Neck Tumors by Stage

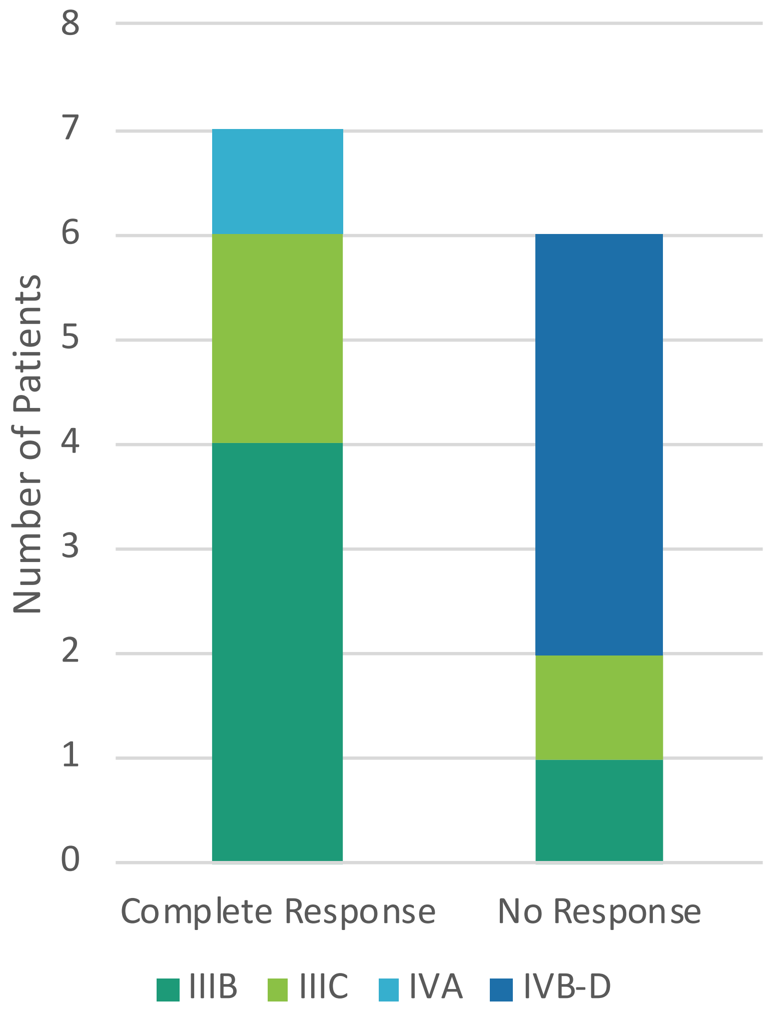

Supplement: Supplementary file 1 — Supplementary file1 (PDF 99 kb) [file 10434_2024_16346_MOESM1_ESM.pdf]
